# Status epilepticus in a patient with intractable epilepsy caused by renal colic due to a ureter stone

**DOI:** 10.1002/iju5.12399

**Published:** 2021-12-06

**Authors:** Koei Torii, Yosuke Ikegami, Maria Aoki, Taiki Kato, Takashi Hamakawa, Tetsuji Maruyama, Takahiro Yasui

**Affiliations:** ^1^ Department of Urology Nagoya City University East Medical Center Nagoya Aichi Japan; ^2^ Department of Urogynecology Nagoya City University East Medical Center Nagoya Aichi Japan; ^3^ Department of Urology Nagoya City University West Medical Center Nagoya Aichi Japan; ^4^ Department of Nephro‐urology Nagoya City University Graduate School of Medical Sciences Nagoya Aichi Japan

**Keywords:** renal colic, seizure‐inducing factors, status epilepticus, ureter stone, ureteroscopic lithotripsy

## Abstract

**Introduction:**

Epilepsy has a variety of seizure‐inducing factors. Epileptic seizures caused by renal colic are extremely rare.

**Case presentation:**

A 22‐year‐old woman with intractable epilepsy was brought to our hospital as an emergency case, because of vomiting and status epilepticus. She had implanted a vagus nerve stimulator in the left anterior chest at the age of 20 years. Computed tomography showed a ureter stone in the right distal ureter. On the second day of hospitalization, ureteroscopic lithotripsy was performed under general anesthesia. The patient’s seizures were controlled to a frequency of once a month or less in the four months after discharge.

**Conclusion:**

We encountered a rare case of the frequency of status epilepticus increased by renal colic due to a ureter stone. Ureteroscopic lithotripsy was effective in controlling the frequency of status epilepticus increased by renal colic.

**Keynote message**

Renal colic due to ureter stones rarely causes status epilepticus. Ureteroscopic lithotripsy may be effective in controlling status epilepticus caused by renal colic due to ureter stones. In patients with epilepsy, renal colic due to ureter stones may cause epileptic seizures, and ureteroscopic lithotripsy can be performed to control status epilepticus.

## Introduction

Convulsive seizures can be triggered by various inducing factors. However, there are no reports of renal colic induced by ureter stones. Herein, we report a rare case of status epilepticus caused by renal colic due to a ureter stone.

## Case presentation

**Fig. 1:**
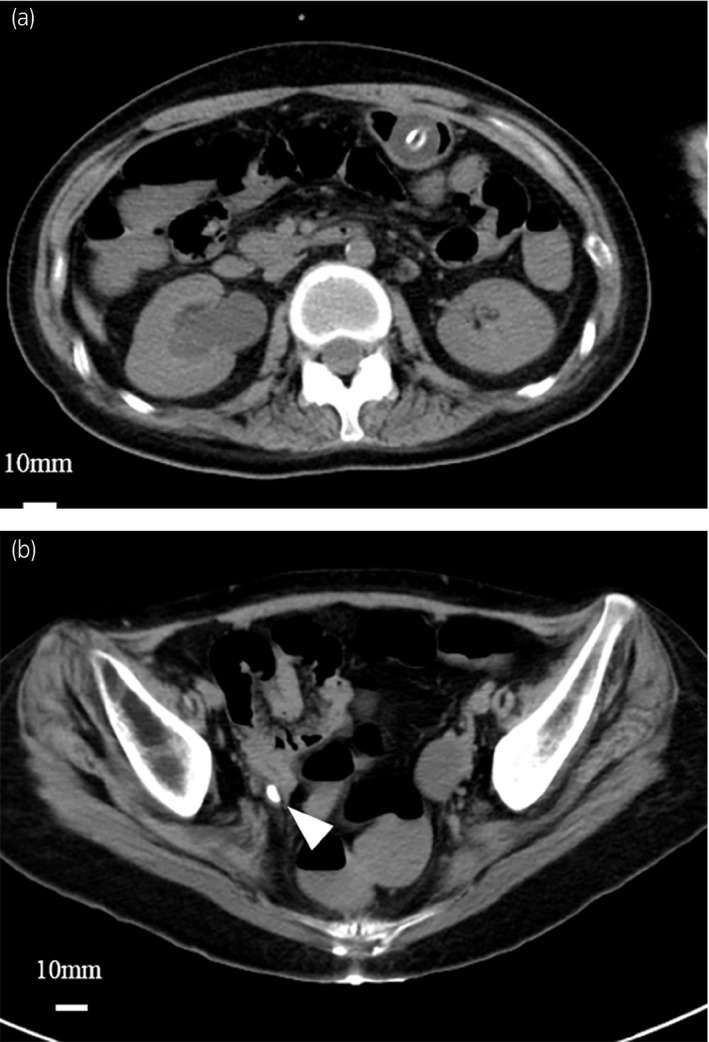
Abdominal unenhanced computed tomography demonstrates (a) hydronephrosis and (b) ureteral stone in the right distal ureter (white arrows).

A 22‐year‐old Japanese woman visited our hospital with a chief complaint of vomiting and convulsive seizures for a month and sudden status epilepticus. She had a history of intractable epilepsy, diagnosed at the age of 18 years and had undergone tracheostomy and gastrostomy management in the same year. It was an intractable epilepsy called acute encephalitis with refractory, repetitive partial seizures. She had been confined to the bed for a long period of time due to intractable epilepsy, and a vagus nerve stimulator had been implanted in the left anterior chest at the age of 20 years. She was taking multiple antiepileptic drugs, none of which were prone to causing the formation of ureter stones. On admission, her blood pressure was 125/93 mmHg, heart rate was 106 beats/min, oxygen saturation was 95% with room air, respiration rate was 20 breaths/min, and temperature was 36.9°C. The Glasgow Coma Scale score was E1VTM4. Her white blood cell count was 6,850 cells/μL, hemoglobin level was 13.8 g/dL, and C‐reactive protein level was 3.1 mg/dL. Urine analysis revealed mild pyuria, but no hematuria. A simple computed tomography scan of the head showed no abnormalities, and chest radiography showed a vagus nerve stimulator implanted in the left anterior chest. A computed tomography scan of her abdomen revealed a ureter stone in the right distal ureter and hydronephrosis (Fig. 1a,b). The diameter of the longest ureter stone was 10 mm. We hypothesized that ureter stone‐induced renal colic was the cause of status epilepticus and performed ureteroscopic lithotripsy under general anesthesia on the second day in the hospital. The ureteral stone component was calcium oxalate. The patient did not have status epilepticus or fever from the day after surgery and was discharged on the 8th day of hospitalization. The patient’s seizures were controlled to a frequency of once a month or less in the 4 months after discharge.

## Discussion

Convulsive seizures have seizure‐inducing factors, and there have been a few systematic reports on the types of their factors.^1^, ^2^

Mattson *et al*. reported certain seizure‐inducing factors for convulsive seizures, namely physical fatigue, mental stress, fever, infection, and neglected medication.^3^, ^4^ In this case, since there was no fever, infection was unlikely to have occurred, and renal colic due to ureter stones was suggested to be the cause of status epilepticus.

Kanemoto *et al*. reported somatosensory stimulation such as rubbing and tapping induced seizures, and Suzuki *et al*. reported that pain or fear of pain‐induced seizures, but there are no reports renal colic‐induced seizures.^5^, ^6^ Therefore, this is the first report of renal colic due to ureteral stones being an inducing factor for status epilepticus.

In this case, a vagus nerve stimulator was implanted in the left anterior chest 2 years previously. Vagus nerve stimulation therapy is a palliative surgical treatment for intractable epilepsy that can suppress convulsive seizures by stimulating the vagus nerve in the neck.

Hassert *et al*. and Ben *et al*. reported that the anticonvulsant effect worked by increasing the release of the neurotransmitters, such as norepinephrine and gamma‐amino butyric acid.^7^, ^8^ In addition, Henry *et al*. and Vonck *et al*. reported that increased cerebral blood flow to the thalamus and cerebral cortex, suppressed convulsive seizures.^9^, ^10^ In this case, the frequency of convulsive seizures decreased to once a month or less for 2 years due to the use of a vagus nerve stimulator. However, the frequency of convulsive seizures gradually increased over the past month, suggesting that convulsive seizures were poorly controlled due to new factors. Renal pain fibers are primarily preganglionic sympathetic nerves that reach spinal cord levels T‐10 to L‐2 through the dorsal nerve roots, and renal swelling with capsular stretching stimulates the sympathetic nervous system.^11^, ^12^ We believe the sympathetic system dominated the parasympathetic system, and the vagus nerve stimulator could not suppress the patient’s convulsive seizures.

It is not uncommon for patients with intractable epilepsy to be confined to the bed for long periods of time. In addition, the risk of stone formation is not low because of the chronic lack of fluid intake caused by gastrostomy management.^13^, ^14^, ^15^ Moreover, since the patient was vomiting, an abdominal computed tomography scan was performed in the emergency room, which enabled the diagnosis of ureter stones. In this case, poorly controlled status epilepticus occurred despite the implantation of a vagus nerve stimulator. Furthermore, since the size of the ureter stone was too large to be passed out spontaneously, ureteroscopic lithotripsy was performed.^16^ As a result, the patient’s seizures were controlled to a frequency of once a month or less after discharge. In addition, increased fluid intake and a moderate frequency of repositioning were considered necessary to prevent stone formation in the future, and advised accordingly.

## Conclusion

We encountered a rare case of the frequency of status epilepticus increased by ureter stone‐induced renal colic. Ureteroscopic lithotripsy was effective in controlling the frequency of status epilepticus increased by renal colic.

## Ethical approval

This study was approved by the Nagoya City University East Medical Center Institutional Review Board (approval number 21‐04‐304).

## Author Contribution

Koei Torii: Conceptualization; Data curation; Formal analysis; Funding acquisition; Investigation; Methodology; Project administration; Writing‐original draft. Yosuke Ikegami: Writing‐review & editing. Maria Aoki: Supervision. Taiki Kato: Supervision. Takashi Hamakawa: Supervision. Tetsuji Maruyama: Supervision. Takahiro YASUI: Supervision; Writing‐review & editing.

## Conflict of interest

The authors declare no conflict of interest.

## Approval of the research protocol by an institutional reviewer board

Not applicable.

## Informed consent

Written informed consent was obtained from the patient's family for publication of this case report and accompanying images.

## Registry and the registration no. of the study/trial

Not applicable.
